# A malignant proliferating trichilemmal cyst arising on the elbow of a man: A case report and review of the literature

**DOI:** 10.1097/MD.0000000000034035

**Published:** 2023-06-23

**Authors:** Dong Yun Lee, Kang Min Han, Jung Soo Yoon

**Affiliations:** a Department of Plastic and Reconstructive Surgery, DongGuk University Medical Center, Seoul, South Korea; b Department of Pathology, DongGuk University Medical Center, Seoul, South Korea.

**Keywords:** malignant proliferating trichilemmal cyst, proliferating trichilemmal cyst, trichilemmal carcinoma, trichilemmal cyst

## Abstract

Trichilemmal cysts (TCs) are common benign cysts that form from the hair follicles in the skin. Proliferating trichilemmal cysts (PTCs) are rare types of TCs characterized by rapid cellular proliferation. Malignant transformation of PTC (MPTC) is a rare adnexal tumor that account for <0.1% of all skin cancers. TCs and PTCs are benign tumors; however, MPTCs grow rapidly and are prone to metastasis.

**Case presentation:**

A 77-year-old man was referred to our hospital with a solitary pinkish mass on his left elbow. Trichilemmal carcinoma arising from a PTC was confirmed through excisional biopsy, and wide excision was performed. One month postoperatively, a cystic mass was observed and was suspected to have local recurrence; however, bursitis was confirmed after excisional biopsy. After 1 year of follow-up, the patient maintained an improvement without recurrence or any other surgical complications.

**Conclusions:**

In addition to being a very rare disease, MTPC occurred in the elbow of a man who does not fit the general etiology; therefore, it is considered an interesting case, and we report this case for academic contribution.

## 1. Introduction

Trichilemmal cysts (TCs), also known as pilar cysts or isthmus-catagen cysts, are common benign cysts that form from hair follicles in the skin.^[1,2]^ The cyst is lined with multiple layers of squamous cells and is typically filled with keratin, a protein found in hair, nails, and skin cells.^[2]^

Proliferating trichilemmal cysts (PTCs) are rare types of TCs characterized by rapid growth and cellular proliferation.^[1–3]^ While a typical TC is a benign and slow-growing lesion, a PTC may grow rapidly and can sometimes become cancerous.

When it becomes cancerous, it is called a malignant transformation of PTC (MPTC).^[3]^ MPTC is rare, accounting for <0.1% of all skin cancers, but it is a potentially aggressive form of skin cancer that arises from PTC. MPTC typically presents as a rapidly growing ulcerating mass with a firm and irregular border. It has the potential to invade the surrounding tissues and metastasize to other parts of the body.

Generally, TC, PTC, and MPTC are more common in elderly women and tend to occur in areas of the skin with a high density of hair follicles, such as the scalp. However, they have also been reported in other locations, including the neck, face, back, chest, and extremities.^[4]^ It is worth noting that PTCs occurring in non-scalp locations are generally rare and may present unusual symptoms, making them more challenging to diagnose.

Here, we report a rare case of MPTC that occurred on the elbow joint with olecranon bursa invasion, which is not a common area, and was treated with wide excision.

## 2. Case presentation

**Figure 1.**
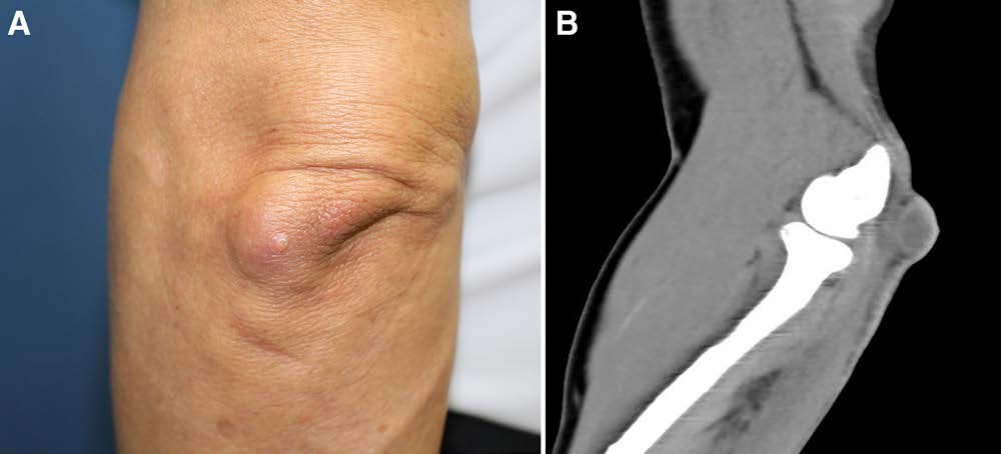
(A) Preoperative clinical photo. (B) The sagittal view of preoperative non-contrast computed tomographic image showing a subcutaneous cystic lesion at the posterior aspect of the radiocapitellar joint without invasion of deep structures.

This study was conducted in conformity with the World Medical Association Declaration of Helsinki, and the protocol was approved by the Institutional Review Board of Dongguk University Medical Center (IRB No.; DUIH 2023-04-002). As per the CARE guidelines, written informed consent was obtained from the patient legal guardian for inclusion of clinical and imaging details for the purpose of publication. A 77-year-old man presented at our hospital with a solitary pinkish mass on his left elbow that had persisted for several years (Fig. 1A). The lesion was a 3 × 2 cm soft and movable cystic mass. He had hyperlipidemia and benign prostatic hyperplasia as underlying diseases and had no specific medical history other than burr hole trephination 7 years prior for subdural hemorrhage. There was no history of repeated trauma or inflammation at the site of the lesion. Preoperative computed tomographic imaging showed a subcutaneous cystic lesion at the posterior aspect of the radiocaptellar joint, without invasion of the deep structures (Fig. 1B).

**Figure 2.**
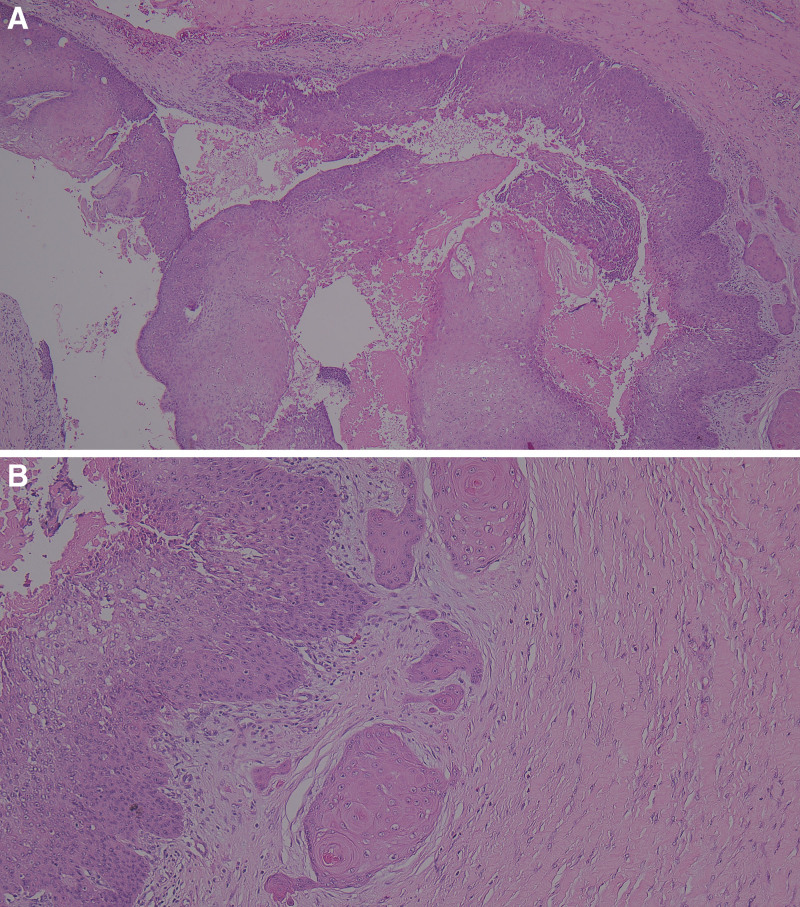
Histologic findings of malignant proliferating trichilemmal cyst. An excisional biopsy showing a fragmented collapsed cystic lesion in the dermis. The cystic lesion is lined with acanthotic stratified squamous epithelium with a central necrotic keratin material. The lining of the squamous epithelium shows abrupt keratinization without a granular cell layer (A, H&E, ×40). In contrast, irregular epithelial nests are found in an area adjacent to the proliferating trichilemmal cyst. The irregular epithelial nests reveal an infiltrative growth pattern and display pilar-type keratinization in the absence of granular cells, which indicates malignant transformation of proliferating trichilemmal cysts (B. H&E, ×100).

An excisional biopsy was performed for the diagnosis. Histologically, an atypical cystic lesion with no granular layer and abrupt necrotic keratinization was observed (Fig. 2A). Additionally, irregular nests with infiltrative growth patterns were observed around cyst walls (Fig. 2B). Through histological findings, it was diagnosed as trichilemmal carcinoma arising from PTCs.

After confirmation of MPTC, a wide excision with a 1-cm safety margin including the bursa was additionally performed. However, a frozen biopsy revealed deep tumor invasion at the base; therefore, additional resection of the deep fascia was performed to secure a sufficient base safety margin. Because anconeus muscle herniation was observed, fascia reconstruction was performed with a Prolene mesh designed to match the fascial defect to prevent further herniation.

**Figure 3.**
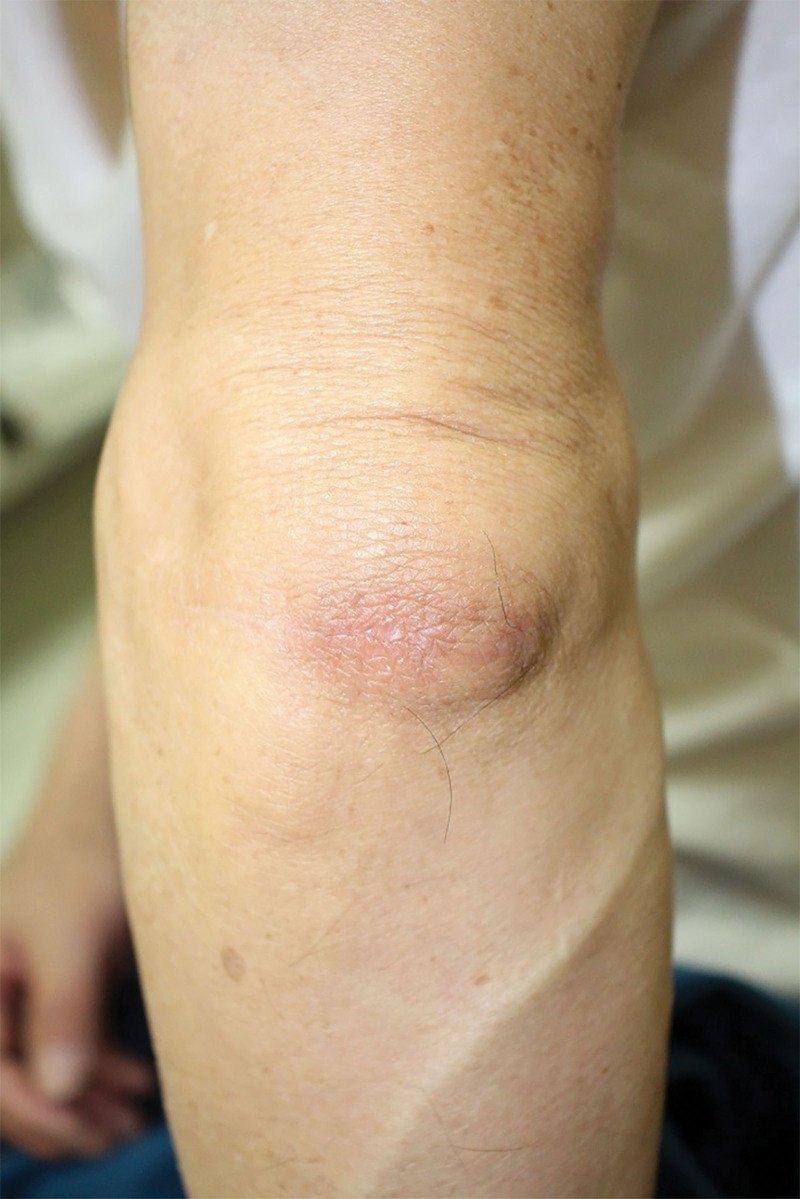
Clinical photo at 1 yr postoperatively.

One month after surgery, the patient complained of a 1.5 × 1.5 cm sized cystic formation at the surgical site. An excisional biopsy was performed again under the suspicion of local recurrence. However, the final diagnosis was bursitis, not recurrence. After 1 year of follow-up, the patient maintained an improvement without recurrence or any other surgical complications (Fig. 3).

## 3. Discussion and conclusions

PTC is a rare tumor that originates from the outer root sheath of hair follicles. This term was first proposed by Jones in 1966 and is also known as Wilson Jones scalp tumor. As of 2018, only 187 cases had been reported.^[1,2]^ It mainly appears in sun-exposed and high hair follicle density areas, and more than 90% of patients have lesions on the scalp. It usually occurs in women in their 60s, with a 4-fold higher occurrence rate than in men. Clinically, it is benign, but can be confused with squamous cell carcinoma because it appears as a red, slightly raised lump that may be accompanied by crusts or ulcers. PTC is a transitional form between TC and a true malignant pilar tumor or MPTC.^[1–3]^

MPTC was first proposed by Saida et al in 1983, and it is known that TC can develop into PTC due to repetitive trauma or inflammation and rarely progresses to MPTC.^[3]^ However, the actual pathogenesis of PTC in MPTC has not been identified. Several studies have suggested that p53 mutation is a key point, as in non-melanoma skin cancer.^[4]^ Takata et al performed DNA analysis of trichilemmal carcinoma arising from the PTC wall and reported that the loss of chromosome arm 17p containing the p53 gene resulted in mutation. Approximately 30 cases of MTPC have been reported until 2004.^[5–8]^ To our knowledge, there have been no case reports of elbow MTPC.

MPTC is clinically difficult to differentiate from PTC and can be confirmed through pathological findings. PTC has characteristic histological features of trichilemmal keratinization, which is an abrupt transition of nucleated epithelial cells to keratinized cells without the formation of a granular layer.^[5,6]^ This keratinization is found primarily in the outer root sheath. Varying levels of inflammation can be present, as indicated by lymphocyte, histiocyte, plasma cell, and occasionally neutrophil and eosinophil levels. In PTC, mitosis is low and mitoses are observed as 0 to 4 per 10 high-power fields; however, if strong mitotic activity or numerous cytonuclear atypia or aneuploidy are observed and irregular infiltrate patterns in the cystic wall are observed, MTPC should be suspected. As keratin pearls may be observed, care must be taken to differentiate them from squamous cell carcinomas. Additional immunohistochemistry, such as CK 16, CD 34, or ki-67, can also confirm the diagnosis.^[7–10]^

A clear guideline for treatment has not yet been presented, but a wide excision with a safety margin of 1 cm arriving at the periosteum is required in MPTC.^[11–14]^ Recently, Moh micrographic surgery achieved good results. However, because the probability of local recurrence between 6 months and 10 years is 3.7%, continuous monitoring is required. Additional postoperative radiotherapy or chemotherapy may be needed if lymph node or distant metastases are present. Several studies have reported improved clinical regression in patients who received postoperative radiotherapy.^[11,12]^ Therefore, patients with high-risk features for local recurrence or metastases may need multidisciplinary treatment plans.

Here, we report a case in which MPTC, a very rare skin cancer, which occurred in the elbow of a man with uncommon etiology. This is a successful case that has maintained improvement without local recurrence through wide excision and bursectomy. To our knowledge, this is the first case of MPTC that occurred in the elbow. Therefore, we report this article for future academic contributions.

## Author contributions

**Conceptualization:** Dong Yun Lee, Jung Soo Yoon.

**Data curation:** Kang Min Han.

**Formal analysis:** Kang Min Han.

**Investigation:** Jung Soo Yoon.

**Resources:** Jung Soo Yoon.

**Supervision:** Jung Soo Yoon.

**Writing – original draft:** Dong Yun Lee.

**Writing – review & editing:** Kang Min Han, Jung Soo Yoon.
